# Promoting sleep health in future healthcare professionals: development and validation of a multi-component lifestyle intervention module for nursing students

**DOI:** 10.3389/fpubh.2025.1698558

**Published:** 2025-11-27

**Authors:** Jing Wang, Ismarulyusda Ishak, Fatin Hanani Mazri, Ching Sin Siau, Fengxue Xin, Arimi Fitri Mat Ludin

**Affiliations:** 1 Taishan Vocational College of Nursing, Taian, Shandong, China; 2 Center for Toxicology and Health Risk, Faculty of Health Sciences, Universiti Kebangsaan Malaysia, Kuala Lumpur, Malaysia; 3 Center for Healthy Ageing and Wellness, Faculty of Health Sciences, Universiti Kebangsaan Malaysia, Kuala Lumpur, Malaysia; 4 Centre for Community Health Studies, Faculty of Health Sciences, Universiti Kebangsaan Malaysia, Kuala Lumpur, Malaysia; 5 College of Biotechnology and Pharmaceutical Engineering, Nanjing Tech University, Nanjing, China

**Keywords:** sleep health, nursing education, lifestyle intervention, module validation, public health

## Abstract

**Background:**

Sleep disturbances are a widespread public health concern among nursing and healthcare students globally, with consequences for academic performance, mental health, and long-term professional competence. Despite evidence linking multiple lifestyle factors, such as physical activity, diet, stress management, and relaxation techniques, to sleep quality, comprehensive interventions integrating these elements remain limited in nursing education. We plan to conduct a pre-registered 4-week pre–post effectiveness pilot and will report effect sizes (Cohen’s *d*, 95% CI).

**Methods:**

This study applied the Sidek Module Development Model (SMDM) to design and validate a multi-component lifestyle intervention module aimed at improving sleep quality among vocational nursing students in China. The module integrated five evidence-based components: physical activity, psychoeducation, music therapy, meditation, and nutrition. Content validity and educational quality were assessed by six independent experts using the Content Validity Index (CVI) and the Template for Evaluating Patient Education Documents (TEMPtED). Reliability and acceptability were further evaluated among 47 nursing students through a Likert-scale questionnaire, with Cronbach’s alpha computed for internal consistency.

**Results:**

The module demonstrated strong psychometric properties. The overall scale-level content validity index (S-CVI/Ave) was 0.985, exceeding the recommended threshold and confirming excellent content validity. All five sub-modules were rated as “Excellent” in educational quality based on the TEMPtED evaluation. Student feedback yielded a Cronbach’s alpha of 0.980, reflecting high internal consistency and suggesting that the module’s structure and materials were coherent and well-received. These results indicate strong preliminary evidence supporting the module’s validity and reliability during the development and validation phase.

**Conclusion:**

This study developed and validated a scientifically robust, educationally feasible, and culturally responsive lifestyle module designed to promote sleep health among nursing students. While the findings demonstrate strong content validity, educational quality, and internal consistency, they represent an initial validation stage rather than evidence of intervention effectiveness. The validated framework provides a solid foundation for future implementation, cross-cultural adaptation, and evaluation studies aimed at examining its impact and long-term sustainability across diverse educational contexts.

## 1 Introduction

For Sleep quality has become a critical public health concern, particularly among university and vocational students in health-related fields. Nursing students are especially vulnerable due to their heavy academic workload, irregular class and clinical schedules, and the psychological demands of caring for patients in training environments. Numerous studies have demonstrated that insufficient or poor-quality sleep negatively affects cognitive performance, attention span, memory consolidation, emotional regulation, and overall mental health (1–3). In the context of nursing education, these impairments may directly compromise professional learning outcomes, reduce clinical competence, and ultimately impact the quality of patient care (4, 5).

Emerging evidence further indicates that lifestyle-related factors play a substantial role in the prevalence of sleep disturbances among young adults. Behaviors such as insufficient or irregular physical activity, poor dietary habits, maladaptive stress management, prolonged screen time, and lack of effective relaxation techniques are strongly correlated with sleep disorders (6–8). Consequently, interventions focusing on a single factor, such as exercise or diet alone, often yield limited improvements. In contrast, multi-component lifestyle interventions that integrate diverse elements such as physical exercise, mindfulness or meditation, music therapy, nutrition education, and psychoeducation have been shown to produce more robust and sustainable effects on sleep quality (9–14).

Despite the growing body of international research, structured intervention programs tailored to the needs of Chinese vocational nursing students remain scarce. Existing sleep health initiatives are often fragmented, overly generalized, or lack systematic guidance and measurable outcomes (15, 16). In contrast, module-based interventions provide unique advantages. The delivery of structured learning materials, step-by-step activities, explicit objectives, and evaluation criteria provides a systematic and replicable framework that can be readily adopted by educators and students alike. Furthermore, the modular design allows for flexibility in adaptation to different educational settings, enhancing scalability and sustainability (17–19).

The Sidek Module Development Model (SMDM) has been widely acknowledged as a rigorous framework for the systematic design, development, and validation of educational modules. Its two-phase structure, which comprises module drafting through expert consultation followed by testing and validation with the target audience, ensures that the final product is theoretically grounded, content-valid, and reliable in real-world applications (20). SMDM has been successfully applied across a range of educational and health-related contexts, further supporting its utility for developing interventions addressing sleep health among nursing students.

Therefore, the present study sought to develop and validate a multi-component lifestyle intervention module intended to enhance sleep quality among vocational nursing students. The specific objectives were: (1) to design and refine the intervention module through an iterative process of expert consultation, (2) to evaluate its content validity and educational quality using expert assessments with the Content Validity Index (CVI) and the Template for Evaluating Patient Education Documents (TEMPtED) instrument, and (3) to assess its internal consistency and acceptability through a structured Likert-scale questionnaire administered to nursing students.

While the study is grounded in the context of Chinese vocational nursing education, its implications extend far beyond national boundaries. Sleep disturbances among nursing and healthcare students are a global challenge, affecting academic performance, clinical competence, and long-term professional well-being. By integrating physical activity, psychoeducation, music therapy, meditation, and nutrition into a structured and culturally sensitive framework, this study provides not only a locally relevant solution but also a potentially adaptable model for nursing and health education programs worldwide. In doing so, it contributes to the broader international effort to improve student health, enhance the quality of professional training, and strengthen the resilience of the future healthcare workforce.

## 2 Methodology

### 2.1 Research design

**Figure 1:**
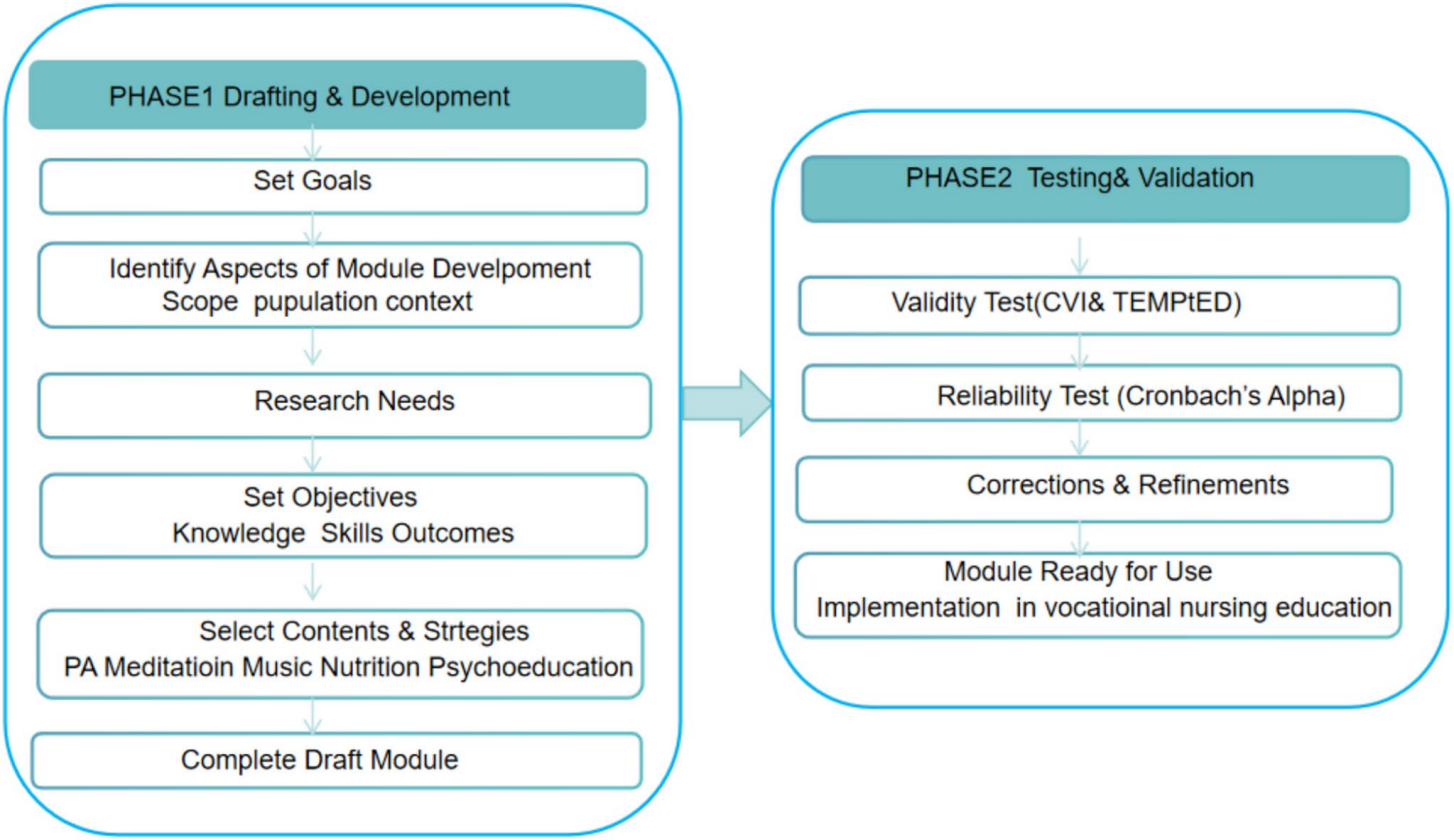
Sidek module development model applied to the sleep quality intervention module.

This study was conducted based on the Sidek Module Development Model (SMDM), which emphasizes a systematic two-phase approach: module development followed by validation. The first phase involved drafting the module by generating preliminary content through a comprehensive literature review and initial expert input, after which the draft underwent multiple rounds of consultation and refinement until consensus was reached. The second phase focused on testing and validation, which comprised two components: expert assessments of content validity and educational quality, and student-based evaluations of reliability and acceptability. This structured design ensured that the developed module was both theoretically grounded and practically feasible for implementation in vocational nursing education. Figure 1 illustrates the overall research process following the SMDM framework.

### 2.2 Participants

The participants in this study comprised both experts and nursing students. For the development phase, six experts were purposively selected to represent a multidisciplinary perspective encompassing sleep medicine, psychology, physical activity, nutrition, music therapy, and meditation. The use of 5–10 experts is recommended in module development and validation studies to balance diversity and manageability (21). A total of six was deemed sufficient to ensure comprehensive evaluation across relevant domains. In the event of disagreement (e.g., a 50:50 split in expert ratings), the item was retained and discussed until a consensus (at least 80% agreement) was achieved through iterative consultation, consistent with the content validation procedures outlined by Grant and Davis Grant and Davis (22).

For the validation phase, another six experts, distinct from those involved in development, were engaged to evaluate the module’s content validity and educational quality. The use of separate panels for development and validation has been recommended to minimize confirmation bias and to enhance the credibility of content validation results (22). The six validation experts included one specialist each in (1) sleep medicine, (2) psychology, (3) nursing education, (4) physical activity, (5) music therapy, and (6) nutrition. All held doctoral or senior professional qualifications and had more than ten years of experience in their respective fields.

To test internal consistency reliability (Cronbach’s *α*) of the module acceptance scale, a sample size was calculated using the single population proportion formula with a margin of error (*δ*) of 0.15, appropriate for reliability pilot studies (27):


n=1.962⋅0.214(1–0.214)0.152≈43

Accounting for 10% attrition, the final recommended sample was 47 students. This number satisfies minimum thresholds for preliminary scale validation and reliability analysis using Cronbach’s alpha (28).

Three instruments were utilized to evaluate the developed module.

First, the Content Validity Questionnaire was distributed to the six validation experts. Each module item was rated on a five-point Likert scale, ranging from 1 (not relevant) to 5 (highly relevant). From these ratings, both the Item-level Content Validity Index (I-CVI) and the Scale-level CVI (S-CVI/Ave) were calculated. Items with I-CVI values above 0.80 were considered acceptable, and an S-CVI/Ave value of 0.90 or above was interpreted as strong evidence of overall content validity.

Second, the TEMPtED instrument (Template for Evaluating Patient Education Documents) was used to assess the educational quality of the module. This tool comprises five dimensions, namely Content, Motivational Principles, Literacy, Layout and Typography, and Graphics. Each item within these dimensions was scored on a four-point scale from 0 (not met) to 3 (fully met). Dimension scores were summed to produce an overall total score, which was then classified according to established thresholds: 49–54 (Excellent), 44–48 (Good), 38–43 (Moderate), and ≤37 (Not suitable).

Third, a Student Acceptability Questionnaire was administered to the 47 nursing students. The questionnaire used a five-point Likert scale to evaluate the clarity, relevance, and feasibility of the module. Responses were used to compute Cronbach’s alpha, a measure of internal consistency reliability.

### 2.3 Procedure

The module development process followed a structured sequence. Based on findings from Phase I and relevant literature, five sub-modules were drafted:

1. Physical activity, addressing exercise type, frequency, and duration.
2. Psychoeducation, focusing on stress and anxiety management and responsible smartphone use.
3. Music therapy, guiding students in selecting and applying therapeutic music.
4. Meditation, providing step-by-step techniques for mindfulness practices.
5. Nutrition, emphasizing dietary balance, caffeine regulation, and practical implementation.

Detailed descriptions of the intervention design and implementation are provided in Supplementary Table S1. Drafts of these sub-modules were presented in workshops involving the six development experts, who reviewed the materials and suggested revisions. Iterative modifications were made until consensus was achieved. In Phase 2, the finalized module was distributed to the validation experts, who assessed content validity and educational quality using the CVI and TEMPtED instruments. Simultaneously, 47 nursing students completed the acceptability questionnaire, which generated data for reliability analysis.

### 2.4 Data analysis

Data analysis followed a multi-step process. I-CVI was defined as the proportion of experts rating an item ≥3 on a five-point scale. Items with I-CVI between 0.78 and 0.89 were discussed, revised for clarity, and retained after consensus was reached. S-CVI/Ave was computed as the mean of all I-CVI values. Discrepancies were resolved when at least 80% agreement was achieved. TEMPtED scores were averaged across six independent raters. For the Content Validity Index, I-CVI was calculated as the proportion of experts rating each item with a score of 3 or higher, while S-CVI/Ave was computed as the average of all I-CVI values across items. For the TEMPtED evaluation, individual dimension scores and total scores were calculated and interpreted according to the classification thresholds described earlier. For the student acceptability data, Cronbach’s alpha was computed to assess internal consistency reliability. Following conventional benchmarks, values ≥0.90 were interpreted as excellent, 0.80–0.89 as good, 0.70–0.79 as acceptable, and values below 0.70 as poor.

## 3 Results

### 3.1 Module development outcomes

The final intervention module was structured around five interrelated sub-modules: physical activity, psychoeducation, music therapy, meditation, and nutrition. Each sub-module incorporated objectives, theoretical justification, recommended strategies, and implementation guidelines. For example, the physical activity module emphasized moderate-intensity aerobic exercise and muscle-strengthening routines, while the nutrition module highlighted balanced dietary intake and caffeine management. The psychoeducation sub-module focused on stress management and smartphone use, the music therapy sub-module guided students in selecting and applying therapeutic music, and the meditation sub-module introduced mindfulness practices with step-by-step exercises.

The module drafting process required several iterative rounds of expert review. Six validation experts were selected based on doctoral or senior clinical qualifications and at least 10 years of experience in sleep medicine, psychology, nursing education, physical activity, or nutrition. These experts were not affiliated with the authors’ institution and had no involvement in module development to ensure independence. The development and validation panels were distinct to reduce confirmation bias. Experts highlighted the importance of aligning the content with the daily schedules of nursing students, who often face irregular sleep–wake patterns due to clinical training. Revisions were made to ensure feasibility, such as recommending 20–30 min of physical activity per day instead of longer sessions. Experts also emphasized cultural appropriateness, for example by suggesting familiar forms of meditation and music genres more acceptable to Chinese students. The final consensus confirmed that the module not only reflected scientific evidence but also met the needs of the specific target population.

This systematic development process aligns with prior research emphasizing that health education modules are most effective when co-designed with multidisciplinary input and tailored to the lived experiences of the intended learners. Thus, the present module represents a significant advancement in bridging theory and practice for sleep health promotion among nursing students.

### 3.2 Content validity (CVI analysis)

The content validity of the module was rigorously evaluated by six independent experts who were not involved in the development stage. Each expert rated the relevance of the module items using a five-point Likert scale. The item-level CVI (I-CVI) values ranged from 0.833 to 1.000, indicating that almost all items were consistently judged as relevant or highly relevant by the majority of experts. The scale-level CVI (S-CVI/Ave) reached 0.985, which is substantially higher than the recommended threshold of 0.80 and suggests excellent overall content validity.

As shown in Table 1, the detailed results highlight the breadth and appropriateness of the module content. The consistently high I-CVI and S-CVI values demonstrate that the expert panel considered the module to be conceptually clear, comprehensive, and reflective of the essential domains required for effective sleep quality interventions.

**Table 1:** Content validity index (CVI) for module items (*N* = 6 experts).

No.	Submodule/Category	Item content	I-CVI
1	Overall module evaluation	The proposed multi-component lifestyle module is likely to improve college students’ sleep quality.	1
2	Physical activity	Physical activity within the lifestyle module is feasible.	1
3	Music	Music within the lifestyle module is feasible.	1
4	Psychoeducation	Psychoeducation within the lifestyle module is feasible.	1
5	Nutrition	Nutrition within the lifestyle module is feasible.	1
6	Meditation	Meditation within the lifestyle module is feasible.	0.833
7	Synergistic effect	Integrating physical activity, psychology, music, meditation, and nutrition into a single module will generate a synergistic effect on sleep quality.	1
8	Relevance of intervention	The proposed interventions are evidence-based and relevant to the target population.	1
9	Importance of physical activity	Regular physical activity is a key factor in improving college students’ sleep quality.	1
10	Aerobic exercise suitability	The aerobic exercise recommended in this module is appropriate for college students.	1
11	Resistance training suitability	The resistance training recommended in this module is appropriate for college students.	0.833
12	Frequency and intensity	The frequency and intensity of the recommended physical activity are appropriate for college students.	1
13	Likelihood of sustained participation	As part of this module, students are likely to continue engaging in physical activity.	1
14	Psychological impact	Addressing psychological factors such as stress and anxiety will significantly improve sleep quality in this population.	1
15	Practicality of psychological intervention	The group-based mental health education included in this module is practical and effective for vocational nursing students.	1
16	Feasibility of psychological support	Students will find psychological support in this module feasible and beneficial.	1
17	Smartphone use guidance	The module will guide students to use smartphones appropriately through group psychoeducation.	1
18	Effect of music therapy	Music therapy is an effective method to enhance relaxation and sleep quality.	1
19	Music selection	The choice of music in the intervention module helps to improve sleep.	1
20	Appropriateness of duration	The recommended duration of the intervention in this module is suitable for improving sleep.	1
21	Formation of music habit	Students are likely to continue using music for sleep and develop it into a habitual practice.	1
22	Positive impact of meditation	Integrating meditation into daily life will have a positive effect on sleep quality.	1
23	Suitability of meditation techniques	The meditation techniques recommended in this module are appropriate for college students.	1
24	Feasibility of daily practice	Practicing meditation for 5 min before bedtime each day is feasible and beneficial for sleep.	1
25	Sustainability of meditation practice	As part of this module, students are likely to adopt and maintain meditation practices.	1
26	Importance of healthy diet	A healthy diet plays an important role in improving sleep.	1
27	Appropriateness of dietary recommendations	The dietary recommendations in this module are scientifically sound and suitable for the target population.	1
28	Feasibility of nutrition implementation	Students will be able to easily implement the nutritional guidelines provided in the module.	0.833
29	Dietary Structure Suggestions	Dietary structure should be reasonably arranged, including intake of staples, vegetables, fruits, proteins, and fats.	1
30	Awareness of coffee’s impact	Coffee has a certain impact on sleep.	1
31	Feasibility of implementation	The overall module is meaningful and feasible to implement in vocational college settings.	1
32	Likelihood of student adoption	The module is likely to be welcomed and adopted by vocational college students.	1
33	Prediction of long-term impact	The module will have long-term positive effects on nursing students’ sleep quality.	1
S-CVI/Ave		**0.985**	

I-CVI, Item-level Content Validity Index; S-CVI, Scale-level Content Validity Index. Acceptable threshold ≥0.80. Items with I-CVI, 0.833 were revised according to expert suggestions and retained, as they remained within acceptable methodological thresholds and did not affect the overall S-CVI/Ave (0.985).

These results align with prior studies in health education, where S-CVI values above 0.90 are generally regarded as strong evidence of content validity. Comparable findings have been reported in module development research related to stress management, physical activity, and nutrition education, further supporting the robustness of the present results. The high content validity established in this study provides confidence that the module’s learning materials and activities are well aligned with its intended objectives and are directly relevant to improving sleep quality among nursing students.

### 3.3 Educational quality (TEMPtED evaluation)

The quality of the developed module was further evaluated through the application of the Template for Evaluating Patient Education Documents (TEMPtED) instrument. This tool measures five key dimensions, namely content, motivational principles, literacy, layout and typography, and graphics. Each dimension is composed of multiple items that were rated on a four-point scale, producing a maximum total score of 54 points for each sub-module. A total of 6 experts independently completed the evaluation, and their scores were averaged to generate the results for each dimension and sub-module.

The evaluation outcomes, as summarized in Table 2, demonstrate that all 5 sub-modules of the intervention achieved mean total scores ranging from 50.0 to 51.5. According to TEMPtED classification thresholds, scores between 49 and 54 indicate excellence, thereby placing every sub-module within the “Excellent” category. The content dimension consistently achieved the highest scores, ranging from 19.0 to 20.3 out of 21, which indicates that experts considered the instructional material to be accurate, comprehensive, and highly relevant to the target population. Motivational principles also received high ratings, with averages between 5.5 and 5.8 out of 6, reflecting that the module successfully incorporated strategies such as goal setting and reinforcement to stimulate student engagement. The literacy dimension was scored between 11.0 and 11.3 out of 12, confirming that the materials were presented using accessible language and appropriate terminology for vocational nursing students. Layout and typography obtained scores of 5.5 to 5.7 out of 6, showing that the overall structure was clear and logically organized, although experts suggested minor adjustments to font size consistency to further enhance readability. The graphics dimension received slightly lower averages, between 8.2 and 8.7 out of 9, with experts recommending the inclusion of more culturally tailored images to strengthen relevance.

**Table 2:** TEMPtED expert evaluation results by sub-module (*N* = 6).

Sub-module	Dimension	Max score	Mean score (Experts)	Classification
Physical activity	Content	21	19	–
	Motivational Principles	6	5.7	–
	Literacy	12	11.2	–
	Layout & Typography	6	5.6	–
	Graphics	9	8.7	–
	Total	54	50	Excellent
Music	Content	21	20	–
	Motivational Principles	6	5.7	–
	Literacy	12	11.3	–
	Layout & Typography	6	5.5	–
	Graphics	9	8.7	–
	Total	54	51.2	Excellent
Psychoeducation	Content	21	20.3	–
	Motivational Principles	6	5.8	–
	Literacy	12	11	–
	Layout & Typography	6	5.7	–
	Graphics	9	8.7	–
	Total	54	51.5	Excellent
Meditation	Content	21	20	–
	Motivational Principles	6	5.5	–
	Literacy	12	11.2	–
	Layout & Typography	6	5.5	–
	Graphics	9	8.5	–
	Total	54	50.7	Excellent
Nutrition	Content	21	20.2	–
	Motivational Principles	6	5.8	–
	Literacy	12	11.3	–
	Layout & Typography	6	5.7	–
	Graphics	9	8.2	–
	Total	54	51.2	Excellent

According to the adapted TEMPtED cut-off criteria for a maximum score of 54, classifications are defined as follows: 49–54 = Excellent, 44–48 = Good, 38–43 = Moderate, and ≤37 = Not suitable.

Despite these minor recommendations, the consistently high ratings across all dimensions confirmed that the module is both scientifically robust and practically applicable. The results provide strong evidence that the educational materials are not only accurate and complete but also user-friendly, visually appealing, and motivational in nature. Achieving “Excellent” scores in TEMPtED across all 5 sub-modules is particularly meaningful because it affirms that the intervention meets the highest standards of educational material design. Prior research in health education has shown that materials classified as excellent in TEMPtED evaluations are more likely to be adopted effectively in educational and clinical environments. Therefore, the current findings strengthen confidence in the feasibility and potential impact of this lifestyle intervention module for improving sleep quality among vocational nursing students.

### 3.4 Reliability analysis (student acceptability)

Reliability testing was conducted with 47 nursing students who evaluated the clarity, relevance, and feasibility of the module through a structured 38-item questionnaire based on a five-point Likert scale. The internal consistency analysis yielded a Cronbach’s alpha coefficient of 0.980, which exceeds the conventional threshold of 0.90 and indicates excellent reliability (Table 3). This result demonstrates that the questionnaire items consistently measured a coherent construct and that student responses were highly internally consistent.

**Table 3:** Reliability analysis of student acceptability questionnaire (*N* = 47).

Sample size (N)	Number of items	Cronbach’s alpha	Interpretation
47	38	0.980	Excellent (≥0.90)

Interpretation based on conventional benchmarks: ≥0.90 = Excellent, 0.80–0.89 = Good, 0.70–0.79 = Acceptable, <0.70 = Poor.

The very high alpha coefficient suggests that the instrument was well-structured and that the items were strongly correlated with each other, reflecting a unified perception of the module’s clarity, relevance, and feasibility. Future module iterations will streamline items to reduce redundancy while maintaining adequate construct coverage and content comprehensiveness. This finding provides strong support for the reliability of the student acceptability questionnaire and reinforces the robustness of the evaluation process. It further indicates that the module was perceived by students as a cohesive and feasible intervention, thereby affirming the consistency of the measurement tool and the stability of the responses.

To further evaluate internal consistency, reliability coefficients were computed for each subscale of the Student Acceptability Questionnaire. Cronbach’s alpha values across subscales ranged from 0.931 to 0.959, demonstrating excellent and consistent internal reliability. Item–total correlations ranged from 0.030 to 0.892, with most items showing moderate to strong positive relationships with the total score. These results confirm the coherence of each subscale and the strong homogeneity of most items, while suggesting that a few items within the Clarity subscale may require refinement in future revisions (Table 4).

**Table 4:** Subscale reliability and item analysis.

Subscale	No. of items	Cronbach’s *α*	Item–total correlation (Min–Max)	Interpretation
Clarity	12	0.931	0.030–0.861	Excellent
Relevance	12	0.955	0.619–0.892	Excellent
Feasibility	14	0.959	0.637–0.870	Excellent

Cronbach’s *α* values above 0.90 indicate excellent internal consistency reliability. All three subscales of the Student Acceptability Questionnaire demonstrated high reliability, suggesting that the items within each dimension consistently measure their intended constructs. The Clarity subscale showed slightly wider variability in item–total correlations (0.030–0.861), indicating that a few items may contribute less strongly to the total scale. Future revisions will review these items to enhance overall measurement precision.

## 4 Discussion

Taken together, the findings indicate that the developed module demonstrates strong psychometric properties across multiple dimensions, including content validity, educational quality, and reliability. The high I-CVI and S-CVI/Ave values confirmed that the content was judged by experts as accurate, comprehensive, and directly relevant to the needs of vocational nursing students (21). Similarly, the TEMPtED evaluation results placed all five sub-modules within the “Excellent” category, confirming that the educational materials met rigorous standards for accuracy, usability, motivation, and visual design (23). These outcomes underscore the importance of combining multidisciplinary expertise and structured evaluation frameworks in developing effective health education modules. We plan to conduct a pre-registered 4-week pre–post effectiveness pilot and will report effect sizes (Cohen’s *d*, 95% CI).

Importantly, this study contributes to the scarce body of literature on sleep health promotion in nursing education. Most prior interventions have focused on single factors, such as physical activity or stress management, with relatively modest outcomes. By contrast, the present study adopted a multi-component approach, integrating physical activity, psychoeducation, music therapy, meditation, and nutrition into a single structured framework. This holistic perspective reflects international best practices in health promotion and highlights the value of addressing sleep health through comprehensive lifestyle interventions (24).

Another important contribution lies in the use of the Sidek Module Development Model (SMDM), which provided a systematic framework to ensure that the module is theoretically grounded, culturally relevant, and educationally feasible. The rigorous validation process enhances its transferability, not only within Chinese vocational colleges but also to other nursing education contexts globally, where students face similar challenges such as heavy workloads, irregular schedules, and poor sleep hygiene (25, 26). By tailoring the intervention to a culturally specific population while ensuring methodological rigor, this study offers a model that can be adapted and scaled across diverse educational and healthcare systems.

These findings are consistent with previous module validation studies emphasizing multidisciplinary collaboration for health education interventions (17, 20, 23). Similar validation frameworks have demonstrated the importance of combining expert consensus and pilot reliability testing in nursing education (21, 24).

From a public health perspective, improving sleep health among nursing students holds significant long-term importance. Nursing students represent the future healthcare workforce, and inadequate sleep is closely associated with diminished academic performance, increased burnout, and reduced clinical competence. Addressing sleep-related issues early in professional education may therefore contribute to improved well-being, enhanced learning outcomes, and ultimately safer patient care.

This study focused on the development and validation of a multi-component lifestyle intervention module designed to promote sleep health among vocational nursing students in China. Guided by the Sidek Module Development Model, the module demonstrated strong content validity, high educational quality, and excellent internal consistency, providing evidence of its scientific soundness and practical feasibility. These findings represent an essential preliminary step toward establishing a structured and culturally responsive framework for sleep health education in nursing programs.

While the study was conducted within a specific national context, the validated framework may serve as a reference for adaptation in other cultural and educational environments. Given that sleep problems among healthcare and university students are a global public health issue, this module offers a potentially adaptable foundation for future implementation and evaluation. Its modular and low-cost structure allows flexible integration into existing nursing curricula and digital learning platforms, supporting future multi-institutional and cross-cultural research on its effectiveness and sustainability.

This pilot was limited to 47 participants from a single vocational nursing college. Findings should therefore be interpreted as preliminary and context-specific. The module framework, while promising, requires replication across multiple institutions, disciplines, and cultural settings to establish generalizability and ensure applicability to broader nursing education contexts.

In conclusion, the present study developed and validated a multi-component lifestyle intervention module designed to promote sleep health among vocational nursing students in China. By establishing strong evidence of content validity, educational quality, and internal consistency, this research provides a solid methodological foundation for subsequent implementation and effectiveness studies. Rather than claiming proven impact, the findings should be viewed as preliminary validation results that demonstrate the feasibility of the module. Future research should focus on multi-institutional pilots, cross-cultural adaptations, and longitudinal evaluations to confirm its effectiveness and sustainability across diverse educational contexts.

## Data Availability

The original contributions presented in the study are included in the article/Supplementary material, further inquiries can be directed to the corresponding author.

## References

[1] Almarzouki AF Mandili RL Salloom J Kamal LK Alharthi O Alharthi S . The impact of sleep and mental health on working memory and academic performance: a longitudinal study. Brain Sci. (2022) 12:1525. doi: 10.3390/brainsci12111525, PMID: 36358451 PMC9688482

[2] Buysse DJ. Sleep health: can we define it? Sleep. (2014) 37:9–17. doi: 10.5665/sleep.3298, PMID: 24470692 PMC3902880

[3] Hyndych A El-Abassi R Mader EC Jr. The role of sleep and the effects of sleep loss on cognitive, affective, and behavioral processes. Cureus. (2025) 17:232. doi: 10.7759/cureus.84232, PMID: 40525051 PMC12168795

[4] Ren W Li W Ji C Kong F Chao L Yang Q . The association between sleep and burnout in psychiatric nurses: a survey from China. BMC Nurs. (2025) 24:639. doi: 10.1186/s12912-025-03238-y, PMID: 40468277 PMC12139159

[5] Vahedi S Davoudi B. The relationship between death anxiety and sleep quality among nurses working in the intensive care unit. Eurasian J Chem Med Pet Res. (2024) 3:129–39.

[6] Darku ED Diyaolu CO. The role of stress, sleep, and mental health in obesity and weight gain. Int Res J Mod Educ Technol Soc. (2025) 6:3216–35. doi: 10.56726/IRJMETS62817

[7] Sharma I Marwale AV Sidana R Gupta ID. Lifestyle modification for mental health and well-being. Indian J Psychiatry. (2024) 66:219–34. doi: 10.4103/indianjpsychiatry.indianjpsychiatry_39_24, PMID: 39100126 PMC11293293

[8] Sun Z Gao X Ren P. The relationship between time anxiety and college students’ sleep quality: the mediating role of irrational procrastination and the moderating effect of physical activity. Front Psychol. (2024) 15:1410746. doi: 10.3389/fpsyg.2024.1410746, PMID: 39027049 PMC11255778

[9] Alnawwar MA Alraddadi MI Algethmi RA Salem GA Salem MA Alharbi AA. The effect of physical activity on sleep quality and sleep disorder: a systematic review. Cureus. (2023) 15:595. doi: 10.7759/cureus.43595, PMID: 37719583 PMC10503965

[10] Aydın Özkan S Başoğul C Karaca T. The effect of psychoeducation-based intervention on sleep quality and anxiety level in third trimester pregnant women: a randomized controlled trial. Curr Psychol. (2024) 43:33170–9. doi: 10.1007/s12144-024-06846-0

[11] Kabalı S Çelik MN Öner N. Associations of nutrition education with diet quality indexes and chronotype: a cross-sectional study. Int J Environ Res Public Health. (2025) 35:2015–27. doi: 10.1080/09603123.2024.2421830, PMID: 39484708

[12] Li Y Tang J Chen G. The effect of meditation-based mind-body interventions on older adults with poor sleep quality: a meta-analysis of randomized controlled trials. Behav Sleep Med. (2025) 23:341–59. doi: 10.1080/15402002.2025.2475911, PMID: 40100065

[13] Morin CM Benca R. Chronic insomnia. Lancet. (2012) 379:1129–41. doi: 10.1016/S0140-6736(11)60750-2, PMID: 22265700

[14] Wang X Feng T Liu S Ruan J. Application of music therapy in improving the sleep quality and mental health of nurses with circadian rhythm sleep disorders caused by work shifts. Noise Health. (2024) 26:294–9. doi: 10.4103/nah.nah_32_24, PMID: 39345067 PMC11539995

[15] Harvey AG Belanger L Talbot L Edelmann K Bastien C Morin CM . Evidence-based psychological treatments for insomnia. Lancet Psychiatry. (2018) 5:975–84.30449712

[16] Lim DC Najafi A Afifi L Bassetti CL Buysse DJ Han F . The need to promote sleep health in public health agendas across the globe. Lancet Public Health. (2023) 8:e820–6. doi: 10.1016/S2468-2667(23)00182-2, PMID: 37777291 PMC10664020

[17] Capezuti E Zadeh RS Brigham MA Dias BA Kim BC Lengetti E . Development and palliative care staff reactions to a sleep regulation educational intervention. BMC Palliat Care. (2022) 21:12. doi: 10.1186/s12904-022-00902-x, PMID: 35062933 PMC8780339

[18] Irish LA Kline CE Gunn HE Buysse DJ Hall MH. Behavioral and psychosocial treatments for insomnia in adults: an update of the 2006 review. Sleep. (2015) 38:217–26.

[19] Roberts K Beckenkamp P Comachio J Castrillon CM Silveira AMC Ho E . The design process and development of MySleepSolutions-A sleep module for a lifestyle self-management app for low back pain. Health Informatics J. (2025) 31:14604582251340548. doi: 10.1177/14604582251340548, PMID: 40380945

[20] Othman MH Johari KSK Amat S. Sidek’s module development model in the Adler marital therapy module. Int J Acad Res Bus Soc Sci. (2023) 13:2023–37. doi: 10.6007/IJARBSS/v13-i10/19102, PMID: 41160198

[21] Polit DF Beck CT. The content validity index: are you sure you know what's being reported? Critique and recommendations. Res Nurs Health. (2006) 29:489–97. doi: 10.1002/nur.20147, PMID: 16977646

[22] Grant JS Davis LL. Selection and use of content experts for instrument development. Res Nurs Health. (1997) 20:269–74. doi: 10.1002/(SICI)1098-240X(199706)20:3<>3.0.CO;2-G, PMID: 9179180

[23] Clayton LH. TemptEd: development and psychometric properties of a tool to evaluate material used in patient education. J Adv Nurs. (2009) 65:2229–38. doi: 10.1111/j.1365-2648.2009.05049.x, PMID: 19686403

[24] Holmbeck GN Devine KA. An author's checklist for measure development and validation manuscripts. Oxford: Oxford University Press (2009). 10.1093/jpepsy/jsp046 PMC2735062 19487232

[25] Brooks SK Greenberg N. Mental health and psychological wellbeing of maritime personnel: a systematic review. BMC Psychol. (2022) 10:139. doi: 10.1186/s40359-022-00850-4, PMID: 35637491 PMC9150387

[26] Leger D Ferini-Strambi L Han F Poyares D Uchiyama M Zee PC. Novel perspective of ‘poor sleep’in public health: a narrative review. BMJ Public Health. (2024) 2:952. doi: 10.1136/bmjph-2024-000952, PMID: 40018608 PMC11816211

[27] Hertzog MA. Considerations in determining sample size for pilot studies. Research in Nursing & Health. (2008) 31:180–91. doi: 10.1002/nur.20247 18183564

[28] Bujang MA Omar ED Baharum NA. A Review on Sample Size Determination for Cronbach’s Alpha Test: A Simple Guide for Researchers. The Malaysian Journal of Medical sciences: MJMS, (2018) 25:85–99. doi: 10.21315/mjms2018.25.6.9 30914882 PMC6422571

