# Supplementary material for: Promoting sleep health in future healthcare professionals: development and validation of a multi-component lifestyle intervention module for nursing students
Source: Front Public Health. 2025 Nov 27;13:1698558. doi: 10.3389/fpubh.2025.1698558 (PMC12695747; doi:10.3389/fpubh.2025.1698558)
Supplement: Supplementary file 1 [file Table_1.DOCX]

**Promoting Sleep Health in Future Healthcare Professionals: Development and Validation of a Multi-Component Lifestyle Intervention Module for Nursing Students**

**Jing Wang ^1,2^,** **Ismarulyusda Ishak^2,*^, Fatin Hanani Mazri^3^, Ching Sin Siau^4^, Fengxue Xin^5^, Arimi Fitri Mat Ludin^3^**

^1^Taishan Vocational College of Nursing, Taishan City,Shandong Province, China

^2^Center for Toxicology and Health Risk, Faculty of Health Sciences, Universiti Kebangsaan Malaysia, Kuala Lumpur, Malaysia

^3^Center for Healthy Ageing & Wellness, Faculty of Health Sciences, Universiti Kebangsaan Malaysia, Kuala Lumpur, Malaysia

^4^Centre for Community Health Studies, Faculty of Health Sciences, Universiti Kebangsaan Malaysia, Kuala Lumpur, Malaysia

^5^College of Biotechnology and Pharmaceutical Engineering, Nanjing Tech University, China.

*Corresponding author

Ismarulyusda Ishak

ismarul@ukm.edu.my

**Table S1. TIDieR Summary Table**
*Template for Intervention Description and Replication (TIDieR) checklist applied to the Multi-Component Lifestyle Intervention Module for Sleep Health.*

| **TIDieR Item** | **Description in This Study** |
| --- | --- |
| **1. Name** | *Multi-Component Lifestyle Intervention Module for Sleep Health (MCLI)* |
| **2. Rationale** | The module was developed to promote sleep health and overall well-being among vocational nursing students by integrating evidence-based lifestyle components, including physical activity, psychoeducation, nutrition, music therapy, and mindfulness meditation. |
| **3. Materials** | Printed module manuals, PowerPoint slides for classroom delivery, audio-guided meditation and music therapy tracks, student worksheets, and weekly sleep diaries. |
| **4. Procedures** | A structured four-week program combining interactive psychoeducation sessions, supervised physical activity routines, guided relaxation and meditation practices, and nutrition awareness activities. Each session included goal-setting, practice, and reflection components. |
| **5. Providers** | Delivered by certified nursing educators, psychologists, and physical activity instructors who were trained in lifestyle intervention facilitation. The delivery team followed a standardized module manual to ensure fidelity. |
| **6. Modes of Delivery** | Primarily in-person classroom sessions supplemented by online materials accessible through a digital learning platform. Participants engaged in both group-based and self-directed learning. |
| **7. Setting** | Conducted at a vocational nursing college in Shandong Province, China, using classroom, fitness room, and dormitory environments for different module components. |
| **8. Frequency and Dose** | Four weekly sessions lasting approximately 90 minutes each, plus daily 10–15 minutes of individual mindfulness or relaxation practice. |
| **9. Tailoring** | The module content was adapted to accommodate students’ academic schedules, clinical rotations, and cultural preferences, ensuring feasibility and engagement. |
| **10. Modifications** | Minor adjustments were made following expert validation, including clarification of exercise intensity, simplification of dietary guidelines, and replacement of certain music examples with culturally preferred tracks. |
| **11. Adherence** | Attendance was recorded for each session. Students completed weekly self-report logs and sleep diaries to track adherence to home practice. |
| **12. Fidelity** | Fidelity was maintained through adherence checklists, instructor supervision, and periodic peer observation to ensure standardized module delivery. |
